# Corrigendum

**DOI:** 10.3109/08958378.2012.655000

**Published:** 2012-01-05

The publisher would like to apologise on behalf of the authors of the following 4 articles published in Inhalation Toxicology.

1. Brorby, Sheehan, Berman, Green, Holm, Re-Creation of Historical Chrysotile-Containing Joint Compounds, Inhalation Toxicology, 20: 1043-1053 (2008).

2. Bernstein, Donaldson, Decker, Gaering, Kunzendorf, Chevalier, Holm, A Biopersistence Study following Exposure To Chrysotile Asbestos Alone or in Combination with Fine Particles, Inhalation Toxicology, 20: 1009-1028 (2008).

3. Bernstein, Rogers, Sepulveda, Donaldson, Schuler, Gaering, Kunzendorf, Chevalier, Holm, The pathological response and fate in the lung and pleura of chrysotile in combination with fine particles compared to amosite asbestosfollowing short-term inhalation exposure: interim results, Inhalation Toxicology, 2010, 22(11) 937-962 (2010).

4. Bernstein, Rogers, Sepulveda, Donaldson, Schuler, Gaering, Kunzendorf, Chevalier, Holm, Quantification of the pathological response and fate in the lung and pleura of chrysotile in combination with fine particles compared to amosite-asbestos following short-term inhalation exposure, Inhalation Toxicology, 2011; 23(7):372-391 (2011).

Since publication of these 4 articles we have had a request to add the following information to the Declaration of Interest section of each paper.

The additional statement reads:

“Georgia-Pacific has not sold chrysotile-containing joint compounds for more than 30 years, but litigation is pending in which individuals claim exposure to the Company's historic products. The articles listed above report on work that Georgia-Pacific commissioned to address issues that have arisen in that litigation. I, Stewart E. Holm, representing Georgia-Pacific, am an author on all four papers. The other authors are consulting experts retained by or on behalf of Georgia-Pacific to conduct the research and prepare the articles. Dr. Donaldson has been listed as potential testifying expert witness by Georgia-Pacific, and Dr. Bernstein has testified as an expert witness for Georgia-Pacific.”

